# FMEA occurrence values for four failure modes occurring using look‐up tables for dose calculations

**DOI:** 10.1002/acm2.13091

**Published:** 2020-11-15

**Authors:** Geoff Nelson, Adam Paxton, Jeremy Kunz, Jessica Huang, Martin Szegedi, Vikren Sarkar, Bill Salter

**Affiliations:** ^1^ Department of Radiation Oncology University of Utah Salt Lake City UT USA

**Keywords:** FMEA

## Abstract

**Purpose:**

For a number of different treatment types [such as Total Body Irradiation (TBI), etc.] most institutions utilize tables from commissioned databooks to perform the dose calculations. Each time one manually looks up data from a large table and then copies the numbers for a manual calculation, there is potential for errors. While a second check effectively mitigates the potential error from such calculations, information regarding the frequency and nature of such mistakes is important to develop protocols and workflows that avoid related errors.

**Methods:**

Five years’ worth of TBI calculations were reviewed. Each calculation was re‐performed and evaluated against the original calculation and original second check. Any discrepancies were noted and those discrepancies were checked to see if the number was the result of misreading from the look‐up table, a typo, copying/skipping partially redundant steps, or rounding/avoiding interpolation. The number of calculations that contained these various types of discrepancies was tallied and percentages representing the frequency of said discrepancies were derived.

**Results:**

All of the discrepancies only resulted in a monitor unit (MU) calculation difference of <1.7%. Typos, looking up wrong values from tables, rounding/avoiding interpolation, and skipping steps occurred in 10.4% (±3.1%), 6.3% (±2.5%), 53.1% (±5.1%), and 4.2% (±2.0%) of MU calculations, respectively.

**Conclusions:**

While all of the discrepancies only resulted in a monitor unit (MU) calculation difference of <1.7%, this review shows how frequently various discrepancies can occur. Typos and rounding/avoiding interpolation are the steps most likely to potentially cause a miscalculation of MU. To avoid direct human interaction on such a large repetitive scale, creating forms that calculate MU automatically from initial measurement data would reduce the incidences that numbers are written/transcribed and eliminate the need to look up data in a table, thus reducing the chance for error.

## 1 INTRODUCTION

This study quantifies common errors that occur during hand calculations. This information is necessary when performing risk analysis such as those outlined in AAPM Task Group Report number 100.^1^ For a number of different treatment types [such as total body irradiation (TBI), Total Skin Electron Therapy, etc.] most institutions utilize tables from databooks to perform the dose calculations. Each time one manually looks up data from a large table and then copies the numbers for a manual calculation, there is potential for errors. While a second check effectively mitigates the potential error from such calculations, it does not tell us how frequently such mistakes occur or the nature of the mistakes. Additionally, this second check is another manual process which itself is prone to error.

This study began in the course of commissioning a form that would automatically calculate the monitor units (MU) for TBI plans. To validate the new form, many previous TBI calculations were checked to see how much they differed from the new form. In this process, it was noticed that there were errors in several old calculations which did not result in a significant difference in total MU, but which were not previously identified as errors. By reviewing these old plans one thing became clear, our second check process was catching gross errors because any plans which had gross differences had been recalculated. However, by automating the process we were now able to identify and quantify failure modes which had been occurring but with a FMEA severity of nearly zero.

Other institutions are encouraged to cross‐examine old data to see if similar rates of “errors" are found. While none of these is of a magnitude that would result in a mistreatment, such information from multiple institutions could help in determining workflows that maximize patient safety and minimize the amount of manual user input.

## 2 MATERIALS AND METHODS

Five years’ worth of TBI calculations were reviewed (62 patient plans). For 47 of these plans, the full calculation sheet from the dosimetrist was available and was reviewed along with the original second check calculation from the physicist. Each calculation was re‐performed and evaluated against the original calculation (47) and original second check (62). In total, 109 calculations were re‐evaluated. Any discrepancies were noted and those discrepancies were checked to assign a failure mode: look up error of misreading from a table, a typo, copying, or skipping partially redundant steps, or rounding/avoiding interpolation. The number of calculations that contained these various types of discrepancies was tallied and percentages representing the frequency of the failure modes were derived.

Determining the failure mode had some ambiguity. Rounding or avoiding interpolating a value from the table was straightforward to determine. If a value from the table was used rather than interpolating the value based on the surrounding table values, it was listed as Rounding/Avoiding Interpolation. Looking up a wrong value from the table was also largely objective. If the value entered matched a value in an immediately adjacent column or row to the value that should have been used it was determined to be an error in looking up the value from the table. Skipped data was obvious, and incorrectly copied data were usually apparent. The typo was assigned if the number entered differed from the correct number by replacing a digit with a number immediately adjacent to the correct number on the keypad. The mistake could have been the result of either a typo or looking up the wrong value in few instances. When this happened, a hierarchy was used. If it could be a wrong lookup value from the table or something else, it was assigned as a mistake in looking up the wrong value. The hierarchy was Looking up the wrong value, Skipped/Copied, Rounding/avoiding interpolation, and finally Typo.

## 3 RESULTS

**Fig. 1:**
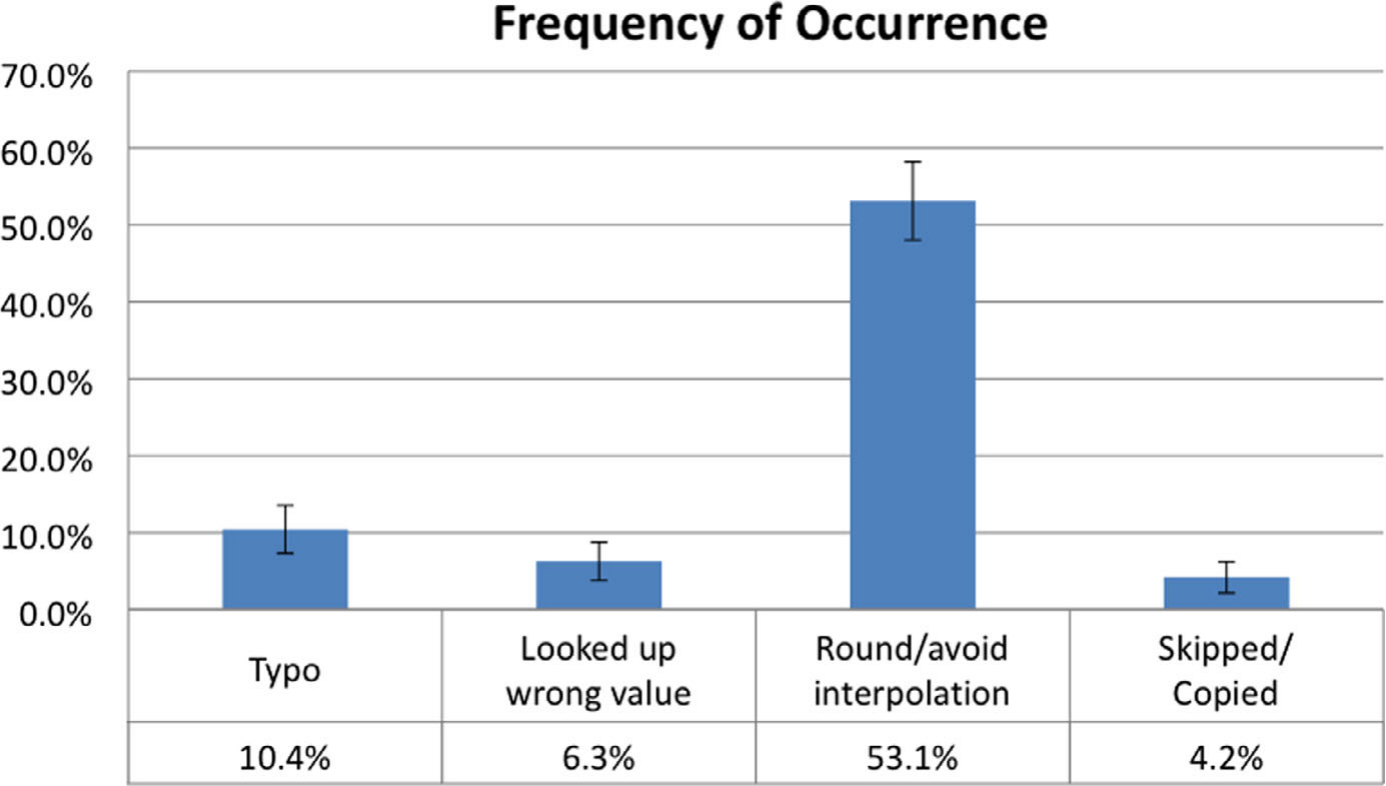
This shows the percentage of total body irradiation dose calculations performed which included an error of each type. Some calculations included multiple types of errors at different parts of the calculation.

All of the discrepancies only resulted in a monitor unit (MU) calculation difference of <1.7%. Typos, looking up wrong values from tables, rounding/avoiding interpolation, and skipping steps occurred in 10.4% (±3.1%), 6.3% (±2.5%), 53.1% (±5.1%), and 4.2% (±2.0%) of MU calculations respectively (see Fig. 1). The results were also divided into the calculations performed by Dosimetrists and the second check calculations performed by physicists.

## 4 DISCUSSION

**Fig. 2:**
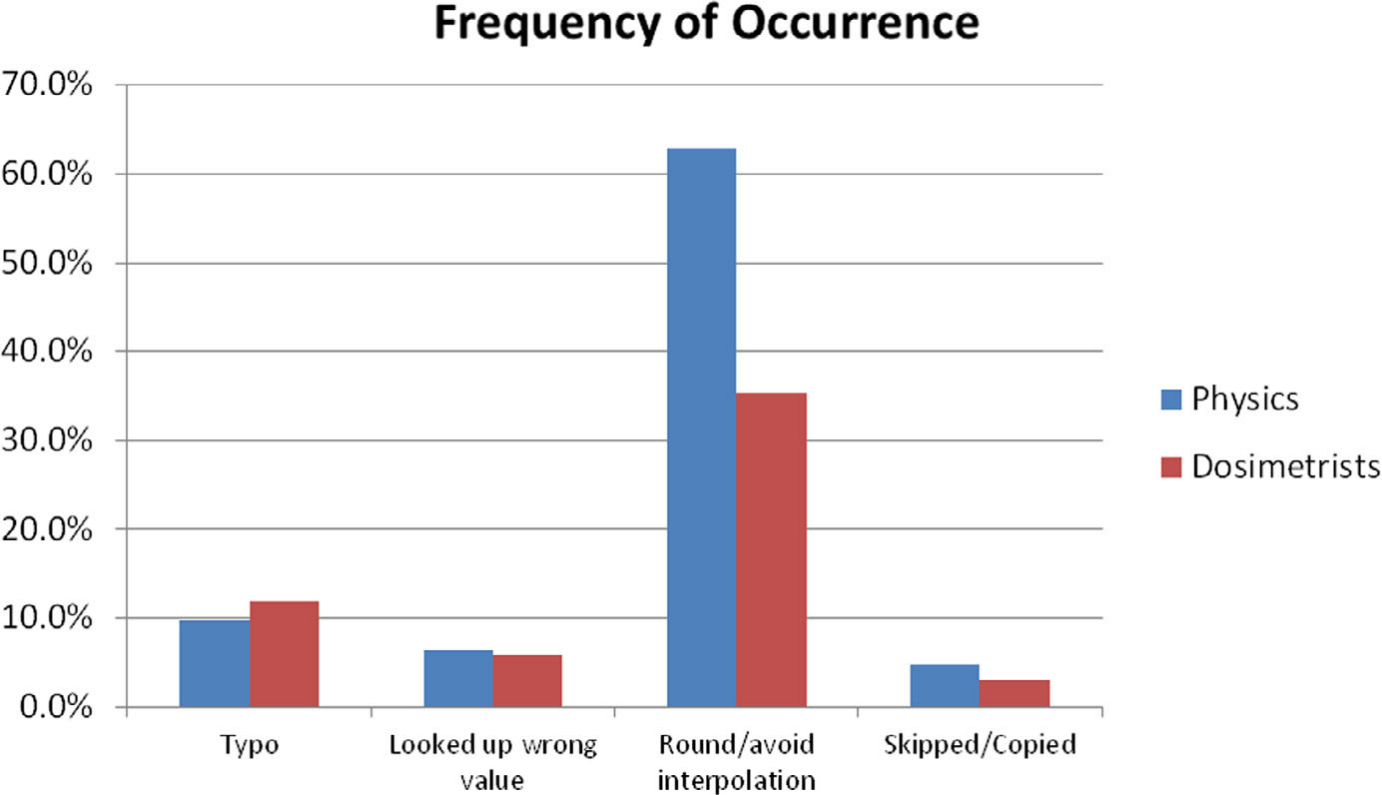
Frequency of Error Types by Group: The percentage of total body irradiation dose calculations performed which included an error of each type by dosimetrists and by physicists.

The frequency of mistakes between physicists and dosimetrists was only statistically different on the frequency of rounding/avoiding interpolating. Of that increase in rounding/avoiding interpolating by physicists, one physicist accounted for 38% of the instances. If that physicist was omitted, the rate was the same between physicists and dosimetrists (see Fig. 2). Given that the difference in MU values between the physicist’s and dosimetrist’s calculations were small enough to consider it as sufficiently accurate, the rounding was likely intentional to save time to in performing the calculation. As such, the category of rounding/avoiding interpolating could very often be an intentional decision and not an error at all. The one physicist who accounted for so many of the rounding/avoiding interpolating instances seems to have intentionally done so to save time knowing that the difference in calculated MU would be very small. It was encouraging to see that the rates for the other categories were consistent across the two groups. Should other institutions perform similar analyses we would expect them to find similar rates for such errors.

Given the nature of the TBI hand calculations performed, it seems likely that similar occurrence rates would be found elsewhere when using look‐up tables for hand calculations. Total body irradiation and total skin electrons (TSE) are the main instances in which hand calculations are performed in this clinic, and they occur with enough regularity that these rates are not likely being affected by this being a rare procedure or because those performing the calculations were less experienced.

This information directly feeds into the likelihood of occurrence (frequently listed as simply O on FMEA analysis). The value for O is an integer from 1 to 10. How this is done is not explicitly stated. Some studies would assign an O value for the Lookup the wrong value, Skipped/Copied, and Typo as presented here in the range of 3 to 6.^2^, ^3^ Others would assign an O value in the range of 9 to 10.^4^, ^5^ These values are high enough that even if one considers the severity (S value) and detectability (D value) to be ideal, these are still going to be worth evaluating.

It should be noted that in reviewing old plans that any problems which may have been caught by our second check calculations were not left in the permanent plan record. It is possible that several of these plans initially had non‐trivial differences between the original plan from the dosimetrist and the second check from the physicist. Based on our available records we were not able to evaluate this. However, if there were additional errors that were caught and corrected, the values presented here could be considered to be a lower bound to the estimate of the occurrence rate.

One possibility is that the physicist performing the second check calculations could have been less careful because their calculated MU was sufficiently close to the dosimetrist’s calculated MU. However, if that were the case we would expect to see more errors from the physicist second check calculations than we see in the dosimetrist’s original plan calculations. Figure 2 indicates that the rates were about the same with the exception of rounding/avoiding interpolation, which as was discussed earlier could be, and very likely was, intentional because the resulting MU was sufficiently close to the planned MU.

## 5 CONCLUSIONS

While all of the discrepancies only resulted in a monitor unit (MU) calculation difference of less than 1.7%, this review shows how frequently various discrepancies can occur. Typos and rounding/avoiding interpolation are the steps most likely to potentially cause a miscalculation. Creating forms that calculate MU automatically from initial measurement data would reduce the number of times numbers are written/transcribed and eliminate the need to consult a look up table, thus reducing the chance for error.

The frequency of occurrences reported here can be used to generate much better informed FMEA analyses of MU calculations using look‐up tables or even more generically for manual entry of data.

## CONFLICT OF INTEREST

No conflict of interest.

## AUTHOR CONTRIBUTIONS

Geoff Nelson conceived, designed, analyzed, and wrote the manuscript. Adam Paxton, Jeremy Kunz, Jessica Huang, Martin Szegedi, Vikren Sarkar, and Bill Salter contributed to interpretation of data, revising, & final approval of the manuscript.
